# Asthma Symptoms Mimicking Myofibroblastic Tracheal Tumor in Pediatric Diagnosis

**DOI:** 10.7759/cureus.74097

**Published:** 2024-11-20

**Authors:** Esperanza Figueroa-Hurtado, Mario J Peña, Arturo Cortes-Telles

**Affiliations:** 1 Respiratory Diseases Clinic, Regional Hospital of High Specialty of the Yucatan Peninsula, Instituto Mexicano del Seguro Social-Bienestar, Merida, MEX; 2 Thoracic Surgery, Hospital Infantil de México Federico Gomez, Mexico City, MEX

**Keywords:** asthma, pediatric, stridor, tracheal tumor, wheezing

## Abstract

Tracheal tumors in pediatric patients are rare, accounting for 2% of all airway abnormalities and 0.2% of all pediatric tumors. Diagnosis is often delayed due to the heterogeneity of presenting symptoms, such as stridor and wheezing, which are frequently misattributed to other conditions. We report the case of a previously healthy nine-year-old male who was diagnosed with an inflammatory myofibroblastic tumor (IMT) following five months of persistent airway symptoms, including cough, biphasic stridor, wheezing, and dyspnea. Despite evaluation by multiple physicians and treatment for presumed asthma, his symptoms did not fully resolve. Imaging studies ultimately confirmed the diagnosis, and surgical resection of the tracheal tumor was performed. In the late postoperative period (12 weeks), the patient continued to experience cough and dyspnea. Given a family history of asthma (father with asthma), spirometry with a bronchodilator was conducted, confirming a diagnosis of asthma alongside IMT. The patient is currently alive and undergoing treatment in Step 2 of the Global Initiative for Asthma (GINA) guidelines. This case highlights the importance of a thorough evaluation in children with persistent stridor and wheezing to rule out underlying tracheobronchial pathologies.

## Introduction

Wheezing and stridor are respiratory sounds caused by turbulent airflow resulting from airway narrowing or obstruction, leading to vibration of the airway walls. Wheezing is characterized by a high-pitched, musical sound, typically occurring during expiration, irrespective of airway caliber. In contrast, stridor is a high-pitched sound during inspiration, produced by accelerated and turbulent airflow narrowed, larger-caliber airways, such as the supraglottic region, larynx, subglottic area, and proximal trachea. Stridor is often a sign of impending airway obstruction and should be treated as a medical emergency. Distinguishing the respiratory phase in which these sounds occur is essential for accurate diagnosis [1,2].

Primary tumors of the larynx, trachea, and bronchi are rare in children, accounting for 2% of airway abnormalities and 0.2% of pediatric tumors [2-5]. Reports in the literature are scarce and primarily consist of limited clinical experiences. Most diagnoses occur during the first decade of life, with younger age at presentation often associated with a poorer prognosis due to disease progression and higher mortality rates [6-8].

Here, we present the case of a nine-year-old male referred to our unit with chronic cough, stridor (initially misdiagnosed as recurrent wheezing), and chest radiographic findings of decreased lucidity in the tracheal tract toward the lower third. A multidisciplinary evaluation ultimately confirmed the presence of a myofibroblastic tracheal tumor.

## Case presentation

A nine-year-old male was referred to our unit with a five-month history of a nonproductive morning cough without other significant aggravating factors. He had been treated multiple times for presumed upper respiratory tract infections, receiving NSAIDs, antihistamines, and antimicrobial regimens.

One month prior to admission, he experienced sudden dyspnea at rest associated with a brief loss of alertness lasting approximately 10 minutes. He was evaluated in the emergency room, diagnosed with an asthmatic crisis, and treated with nebulized short-acting beta-2 agonists before being discharged with symptomatic management.

Twenty days after this event, he developed severe dyspnea at rest accompanied by persistent stridor. He presented to the emergency department of a general hospital with worsening dyspnea characterized by polypnea, increased work of breathing, intermittent stridor, cough, and chest pain. Treatment was again directed toward the presumed diagnosis of an asthmatic crisis, involving intramuscular systemic steroids and nebulized short-acting beta-2 agonists. He was discharged a few hours later following symptomatic improvement.

Despite these interventions, the patient’s dyspnea and stridor persisted, with the stridor gradually becoming louder and audible at a distance. He was subsequently evaluated by pediatric allergology and otorhinolaryngology specialists, who reinitiated treatment with short-acting beta-2 agonists, but his symptoms showed no improvement. A chest X-ray revealed decreased tracheal lucidity in the distal third, prompting his transfer to our unit for further evaluation, including a CT scan of the chest.

**Figure 1:**
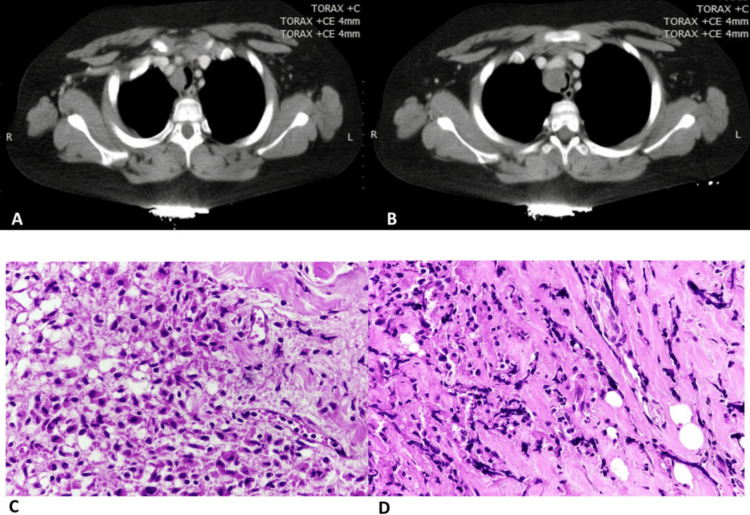
CT scan and histopathological images (A, B) Chest CT scan displaying a homogeneous tumor located in the lower third of the trachea on the right side, compressing 90% of the tracheal lumen. (C, D) Histopathological analysis reveals a benign neoplasm composed of spindle cell proliferation-forming clusters, interspersed with inflammatory cells, including lymphocytes, plasma cells, and macrophages. Extensive areas of both old and recent necrosis are observed, associated with macrophages exhibiting vacuolated cytoplasm.

The chest CT scan revealed a heterogeneous tumor located at the level of the lower right paratracheal third, approximately 2 cm above the main carina. The tumor caused extrinsic compression, obstructing 90% of the tracheal lumen (Figure 1).

With these findings, the patient was evaluated by the pediatric thoracic surgery team, who planned tumor resection via median sternotomy with support from extracorporeal circulation. Tumor resection and tracheal plasty were performed, involving the removal of five tracheal rings and termino-terminal anastomosis of the trachea. The postoperative period was uneventful.

Histopathological analysis confirmed the diagnosis of an inflammatory myofibroblastic tumor (IMT; Figure 1C, 1D). The patient was discharged after a 12-day hospital stay with arrangements for outpatient follow-up.

Four months post-surgery, pulmonary function was assessed due to persistent cough and mild dyspnea during moderate physical activities. Spirometry revealed a moderate airflow obstruction (FEV1/FVC = 0.70, FEV1 = 71% predicted, z-score = -2.61), with a significant 16% improvement in FEV1 after bronchodilator administration. However, the post-bronchodilator test indicated a preserved ratio impaired spirometry pattern (FEV1/FVC = 0.81, FEV1 = 79% predicted, z-score = -1.86), confirming the coexistence of asthma.

Currently, the patient remains alive and in his first 12 months post-surgery without complications or tumor recurrence. He is under treatment with low-dose inhaled corticosteroids (Global Initiative for Asthma (GINA) Step 2), achieving good asthma control without exacerbations.

## Discussion

Acute or subacute stridor is a medical emergency due to its strong association with airway obstruction, which can be severe and potentially fatal. Stridor caused by the narrowing of the intrathoracic airway can produce a bimodal respiratory sound (inspiratory and expiratory) with accompanying wheezing, leading to diagnostic confusion and delays [6-8]. In such cases, patients are often evaluated by multiple physicians (pediatricians, general practitioners, and specialists) who may focus on the apparent medical history - in this case, a paternal history of asthma. However, persistent symptoms despite established treatment necessitate consideration of early differential diagnoses [9].

In pediatric patients with stridor, a thorough clinical evaluation is crucial, encompassing a detailed medical history, a complete physical examination, imaging studies, and, when available, airway endoscopy. This approach is particularly important in cases of biphasic stridor, which may indicate conditions such as foreign body aspiration, croup, epiglottitis, congenital vascular malformations, or tracheal stenosis. Such a systematic assessment enables timely diagnosis and treatment, reducing the risk of complications and long-term sequelae associated with airway obstruction.

Recent publications from airway tumor research groups in Chile and Italy highlight the need for a more detailed classification of primary solid tracheobronchial tumors in pediatrics [9]. Airway tumors are categorized as extrinsic or intrinsic. Extrinsic tumors originate outside the tracheobronchial tree and can compress or infiltrate the airway based on their anatomical location in the cervical region, thorax, or mediastinum. Of these, 70% are malignant, and 30% are benign. Mediastinal tumors are the most frequently reported extrinsic tumors, with neurogenic origins (38%), lymphomas (18%), sarcomas (15%), and germ cell tumors (8%) being the most common. Conversely, intrinsic airway tumors are predominantly benign (98%), with only 2% being malignant. However, of all intrinsic solid tumors, 62% are malignant [10].

The rarity of tracheobronchial tumors in children, combined with their nonspecific respiratory presentations and the low likelihood of clinical suspicion, often delays diagnosis. Obstructive symptoms typically become evident only when the airway lumen narrows by 50% or more. Common clinical manifestations include stridor, chest pain, and hemoptysis [11].

IMT, also known as an inflammatory pseudotumor, is a rare pseudoneoplastic lesion most commonly found in the lung but has been reported in various extrapulmonary sites [9]. The natural course of IMT is not well understood. Although it is relatively common in pediatric patients, reports of IMT involving the trachea are exceedingly rare, accounting for only 0.04-0.07% of all respiratory tract neoplasms. In approximately 50% of pediatric cases, the tracheobronchial tree and lungs are involved. Symptoms such as dyspnea, cough, stridor, and wheezing typically resolve after tumor resection [12-14].

In our patient, 16 weeks after tumor resection and tracheoplasty, the persistence of symptoms alongside a family history of asthma prompted post-bronchodilator spirometry, which confirmed the coexistence of IMT and asthma. This diagnosis was supported by elevated serum immunoglobulin E levels (654 IU/dL; reference cutoff 88 IU/dL) and eosinophilia (390 cells × 10³/mcL). Our literature review did not identify previous reports of simultaneous asthma and IMT diagnoses. While asthma is the most common non-infectious chronic respiratory disease in pediatrics, its coexistence with such a rare tumor is unusual.

The patient remains alive and continues treatment for asthma control, with regular follow-up for the tracheobronchial tumor, including chest CT scans.

## Conclusions

The tracheal myofibroblastic tumor is a benign neoplasm with a low prevalence in children, which may explain the delay in diagnosis in our patient. The initial symptoms included wheezing and mild dyspnea, followed by intermittent biphasic stridor that progressively worsened and became continuous. Stridor, whether inspiratory or biphasic, necessitates a systematic approach, as its presence indicates potential airway obstruction, posing a life-threatening risk to the patient.

Evaluating such cases requires a thorough clinical history, including details about symptom onset, duration, progression, and accompanying signs. Additionally, the patient's medical history should be reviewed to guide the identification of potential causes. In medical facilities equipped with the necessary resources, airway endoscopy (e.g., bronchoscopy) is recommended when an obstruction is suspected to confirm the diagnosis. Recurrent symptoms, such as cough, dyspnea, and wheezing, that respond poorly to standard treatments (e.g., bronchodilators or inhaled steroids) and worsen despite intervention should prompt reconsideration of the diagnosis. A comprehensive evaluation, including pulmonary function tests, imaging studies, and bronchoscopy, is essential to determine the underlying etiology, particularly in cases with rapidly worsening symptoms and sudden-onset stridor.
